# Impact of the COVID-19 pandemic on the incidence of central precocious puberty: A PRISMA-ScR-COMPLIANT scoping review

**DOI:** 10.20945/2359-4292-2024-0300

**Published:** 2025-05-20

**Authors:** Amanda Veiga Cheuiche, Marcelo Garroni Teixeira, Candice Moro, Gustavo Guimarães, Liliane Salvador, Mauro Antônio Czepielewski, Leila Cristina Pedroso de Paula, Sandra Pinho Silveiro

**Affiliations:** 1 Programa de Pós-graduação em Ciências Médicas: Endocrinologia, Universidade Federal do Rio Grande do Sul, Porto Alegre, RS, Brasil; 2 Serviço de Endocrinologia, Hospital de Clínicas de Porto Alegre, Porto Alegre, RS, Brasil

**Keywords:** Precocious puberty, COVID-19, scoping review, incidence

## Abstract

Puberty is a biological maturation process that involves genetic, nutritional, environmental, ethnic, and lifestyle factors. During the coronavirus 2019 (COVID-19) pandemic, an increase in referrals for central precocious puberty (CPP) assessment was observed in clinical practice. The aim of this review was to evaluate the incidence of CPP in different countries before and during the COVID-19 pandemic. A PRISMA-ScR-compliant scoping review was performed in the MEDLINE and Embase databases using “puberty” and “COVID-19” as search terms. Exclusion criteria were an identifiable organic cause of CPP, genetic disorders or peripheral precocious puberty. The study was registered in OSF. A total of 26 studies with participants from 11 countries were included. Twenty-five studies found a 1.3- to 5-fold increase in the incidence of CPP in girls. In boys, 4 studies found no significant difference in the number of cases, 3 studies found a 2.8- to 3.4-fold increase, and 1 study detected a 75% decrease. Twelve studies reported an increase in the use of electronic devices, sedentary lifestyles, higher Z-scores for weight and body mass index, increased sleep disturbances, and a lower age at the onset of puberty. Seven studies found no significant differences in clinical and laboratory parameters between the pandemic and pre-pandemic periods. There was an increase in the incidence of precocious puberty among girls during the COVID-19 pandemic. This finding was not consistently observed in boys. Increased screen time, reduced physical activity, psychological stress, changes in diet and sleep habits, and the direct effects of SARS-CoV-2 may have caused these results.

## INTRODUCTION

The process of puberty involves genetic, nutritional, environmental, ethnic, and lifestyle factors (^1^,^2^). A change in the pattern of pituitary gonadotropin secretion serves as the hormonal trigger for the onset of puberty, but the complete mechanisms underlying this process are not fully understood (^1^,^3^). Factors such as adipose tissue hormones, the gastrointestinal axis, adrenal androgen production, endocrine disruptors, fetal life, and psychosocial stress may influence puberty (^3^). In addition, the progression of puberty requires the interplay of various genetic and epigenetic factors, as well as an intact and normally functioning hypothalamic-pituitary-gonadal (HPG) axis (^4^).

The classic definition of precocious puberty is the development of secondary sexual characteristics before the age of 8 years in girls and before the age of 9 years in boys (^2^). It is classified as central precocious puberty (CPP) when there is premature maturation of the HPG axis, primarily marked by altered hypothalamic GnRH pulsatility. In contrast, peripheral precocious puberty (PPP) occurs due to excessive secretion of sex hormones from a tumoral or exogenous source, or as a result of a genetic disorder, independent of gonadotropin secretion (^1^). Evidence suggests that genetic causes may contribute to the occurrence of CPP and PPP, but they are more frequently associated with CPP. Studies have shown that some rare mutations in the genes *MKRN3*, *DLK1*, and *MECP2* may be involved in the occurrence of CPP (^5^-^7^). Precocious puberty occurs more frequently in girls (15-20 girls for every boy), but the incidence of CPP varies widely across geographical regions (^8^,^9^).

Since the second trimester of 2020, after the COVID-19 pandemic began, several centers have observed a significant increase in the number of appointments for evaluation of precocious puberty (^10^). Different mechanisms have been proposed to explain this phenomenon, including increased screen time and nutritional and psychological factors (^10^,^11^). In this context, this study aimed to evaluate the incidence of CPP before and during the COVID-19 pandemic in different countries by means of a scoping review of the literature.

## METHODS

### Protocol and registration

This scoping review follows the recommendations of the PRISMA Extension for Scoping Reviews (PRISMA-ScR) protocol (^12^,^13^) and has been registered in the OSF (osf.io/27pzj).

### Eligibility criteria

Studies comparing the incidence of CPP before and during the COVID-19 pandemic were included. Exclusion criteria were an identifiable organic cause of CPP, genetic disorders, or PPP. There were no language or date restrictions; articles written in languages other than English, Portuguese, and Spanish were considered eligible if they contained sufficient English-language information in the abstract, tables, and figures.

### Sources of information, search strategy, and selection process

A systematic search of the MEDLINE (via PubMed) and Embase databases was conducted from the inception of the COVID-19 pandemic (specifically, December 2019) to December 2023. Comprehensive search queries included descriptors (MeSH and Emtree) based on the terms “puberty” and “COVID.” The following electronic search strategy was used: (“puberty” [MeSH Terms] OR “puberty” [All Fields] OR “puberties” [All Fields]) AND (“sars cov 2” [MeSH Terms] OR “sars cov 2” [All Fields] OR “covid” [All Fields] OR “covid 19” [MeSH Terms] OR “covid 19” [All Fields]) for MEDLINE and (‘puberty’/exp OR puberty) AND (‘puberty’/exp OR puberty) AND (‘coronavirus disease 2019’/exp OR ‘coronavirus disease 2019’) for Embase. The final search results were exported to EndNote and duplicates were removed. Two independent reviewers (A.V.C. and C.M.) assessed records for inclusion based on titles and abstracts. Abstracts that did not meet the inclusion criteria or that met the exclusion criteria were discarded. The remaining records and those whose abstracts did not provide sufficient information to decide on exclusion were selected for full-text evaluation, which was carried out independently by the same reviewers. A third reviewer (S.P.S.) resolved disagreements.

### Data collection and items

The investigators (A.V.C., C.M., M.G.T., G.G., L.S.) analyzed the selected studies and extracted data using a standardized system. The following information was obtained: first author, year of publication, country, sample size, period of evaluation, number of CPP cases in the pre pandemic and pandemic periods, and clinical and laboratory data evaluated.

### Effects measures and synthesis of results

The information extracted from the included studies was summarized in tables.

## RESULTS

**Figure 1:**
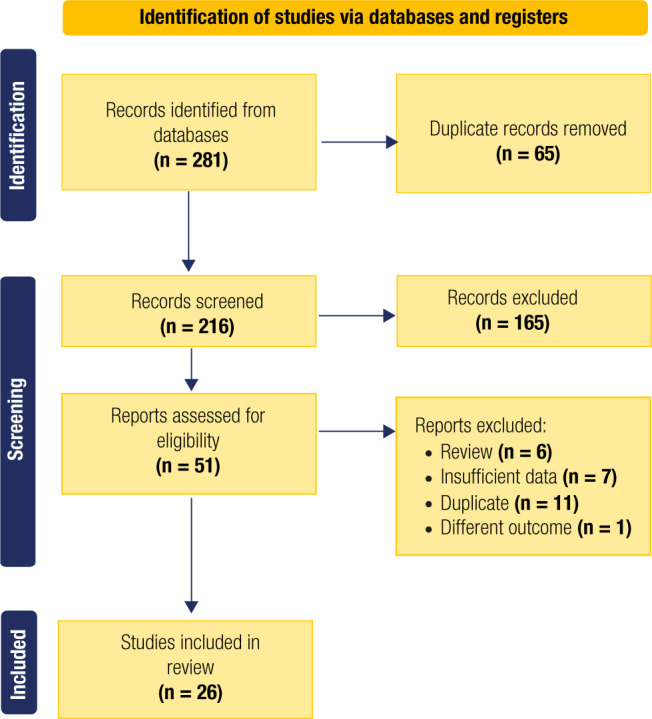
Study flowchart for the scoping review process.

The search strategy identified 281 citations, of which 216 remained after the removal of duplicates. We excluded 165 articles after screening the titles and abstracts based on predefined exclusion criteria, leaving 51 studies for full-text evaluation. Ultimately, 26 studies were selected for the scoping review after further application of the exclusion criteria. **Figure 1** shows a flowchart of the study selection process.

The selected articles were published from 2020 to 2023 and included girls from 11 countries: Italy; Turkey; China; Spain; Argentina; South Korea; Japan; Lebanon; Brazil; the United States; and India (^14^-^39^). The majority of studies were identified in Italy (n = 10) and Turkey (n = 4), while the other countries had one or two articles each. The largest sample size was that of the study carried out in South Korea, which used the national population census (^21^). **Tables 1-4** shows the main characteristics of the studies.

**Table 1:** Positive findings in increased cases of CPP

Reference	Study Method	Country	Population	Exposure/Case	Comparison/Control	Sample Size, n	Outcome			
							Group	Control	Exposure	Observed changes
Verzani M, et al. 2021 (^15^)	Retrospective analysis of consultations in outpatient clinic of Endocrinology	Italy	Patients referred to Endocrinology Unit for suspected precocious puberty	March to September of 2020	March to September of 2019	302 females	Suspected precocious puberty consultations	87	215	2.47-fold increase in consultations for suspected PP
						15 males		9	6	No difference observed in males;
Jimenez A, et al. 2022 (^17^)	Case-Control Study	Spain	Patients < 14 yo referred to Endocrinology Unit from primary care	March to December of 2020	March to December of 2019	133; no info on sex	Percentage of endocrinology referrals attributed to precocious puberty	45/598 (7.5%)	87/471 (18.5%)	Risk Ratio of 2.46
Barbieri C, et al. 2022 (^18^)	Case-Control Study	Italy	Girls referred to Tertiary Center for different forms of precocious puberty	March 9th of 2020 to April 30th of 2021	January 1st of 2019 to March 8th of 2020	154 females	Incidence of CPP in a tertiary center (endocrinology referrals)	9/67 (13.43%)	17/87 (19.54%)	Risk Ratio of 1.45
Chen Y, et al. 2022 (^19^)	Cross-sectional study (Retrospective cohort)	China	Girls admitted in endocrinology ward	2020 (January to December)	2016, 2017, 2018 and 2019 (January to December)	2,802,387; no info on sex	Frequency of precocious girls admitted in endocrinology ward	106/698 (15.18%)	372/1161 (32.04%)	Risk Ratio of 2.10 (only girls)
			All consultations in outpatient clinic				Incidence of precocious girls in outpatient clinic	6,547/1,611,699 (0.4%)	9270/1,188,829 (0.77%)	Risk Ratio of 1.91 (only girls)
Chioma L, et al. 2022 (^20^)	Retrospective cohort	Italy	Patients investigated in five Italian tertiary centers of Pediatric Endocrinology	March to September of 2020	March to September of 2019		Subjects referred for suspected precocious puberty	152	338	2.22-fold increase in referrals.
						490 (22)	Incidence of CPP in tertiary centers	37/140 (26.42%)	135/328 (41.15%)	Risk Ratio 1.55 in females;
								-	-	No difference in males
Itani A, et al. 2022 (^23^)	Retrospective cohort	Lebanon	Medical records of Lebanese patients referred to pediatric endocrinology clinics in multiple Lebanese regions	March of 2020 to February of 2021	2018 and 2019	1380 females	Incidence of CPP diagnosis in endocrinology referrals	4/964 (0.41%)	19/416 (4.6%)	Risk Ratio of 11.21
							Incidence of accelerated puberty diagnosis in endocrinology referrals	8/964 (0.83%)	6/416 (1.44%)	Risk Ratio of 1.73
Mondkar SA, et al. 2022 (^25^)	Case-Control Study	India	Single center, retrospective study, wherein data at the first visit to a tertiary level pediatric endocrinology center were mined	March of 2020 to September 2021	September of 2018 to February of 2020	7261; no info on sex	Incidence of CPP diagnosis in endocrinology referrals	42/4208 (0.9%)	130/3053 (4.2%)	Risk Ratio of 4.66 in females
								2/4208 (0.1%)	06/3053 (0.2%)	Risk Ratio of 2 in males
Mutlu GY, et al. 2022 (^26^)	Case-Control Study	Turkey	Girls who had been referred to pediatric endocrinology clinics due to precocious puberty (retrospective evaluation of medical records)	March of 2020 to September of 2020	March of 2019 to March of 2020	359 females	Incidence of CPP diagnosis in endocrinology referrals due to precocious puberty	19/214 (8.8%)	42/145 (28.9%)	Risk Ratio of 3.28 during first 6 months of pandemic
Peinkhofer M, et al. 2022 (^29^)	Cross-sectional study	Italy	Children and adolescents performing a stimulation test in a tertiary hospital (retrospective analysis)	January to December of 2020	January to December of 2019	83 females	Diagnosis of CPP using LHRH test from suspected cases	16/38 (42.1%)	26/45 (57.7%)	Risk Ratio of 1.37 in females; p = 0.03
						19 males		8/10 (80%)	2/9 (22.2%)	Risk Ratio of 0.27 in males; p > 0.05
Trujillo MV, et al. 2022 (^31^)	Retrospective cohort	USA	Patients referred to Endocrinology Unit for evaluation	May of 2020 to April of 2021	May of 2018 to April 2019	4601, no info on sex;	Incidence of CPP diagnosis in endocrinology referrals	28/2340 (1.2%)	62/2261 (2.8%)	Risk Ratio of 2.33
Umano GR, et al. 2022 (^32^)	Retrospective cohort	Italy	Female patients that attended the outpatient clinic of pediatric endocrinology because of CPP	April of 2020 to April of 2021	2017 to 2020 (April to April)	69 females	Absolute increase in number of CPP case	11†	35	3.18-fold increase in the period.
							CPP incidence rate	2%	5%	Risk Ratio of 2.5
Chioma L, et al. 2023 (^30^)	Retrospective cohort	Italy	Patients consulting for suspected precocious or early puberty in outpatient clinic	March to September of 2020	March to September of 2019	550 females	Prevalence of suspected precocious puberty among endocrinology consultations	78/1260 (6.2%)	202/747 (27%)	Prevalence Ratio of 4.35 in 2020.
							Incidence of CPP diagnosis in endocrinology referrals	18/78 (23%)	86/202 (42.57%)	Risk Ratio of 1.85 during the lockdown.
Goffredo M, et al. 2023 (^32^)	Retrospective cohort	Italy	Patients referred to Endocrinology Units	March of 2020 to February of 2021	March of 2019 to February of 2020	2532, no info on sex.	Incidence of CPP diagnosis in endocrinology referrals	34/1469 (2.3%)	45/1063 (4.2%)	Risk Ratio of 2.12
Benedetto M, et al. 2023 (^36^)	Retrospective cohort	Argentina	Patients diagnosed with ICPP and receiving treatment with GnRHa	January to December of 2020 and 2021	January to December of 2018 and 2019	8874 (0)	Annual incidence of CPP	3,95%	10.9%¥	Risk Ratio of 1.64-3.92.
Matsubara K, et al. 2023, (^37^)	Retrospective cohort	Japan	Patients referred for suspected precocious puberty and diagnosed with CPP	April of 2020 to April of 2021	April of 2019 to April of 2020	1292 females	Incidence of CPP diagnosis in endocrinology referrals	28/248 (11.29%)	51/271 (18.81%)	Risk Ratio of 1.66 in females.
						1051 males		5/204 (2.4%)	12/170 (7.05%)	Risk Ratio of 2.93 in males.
Fava D, et al. 2023 (^38^)	Retrospective cohort	Italy	Health records of girls referred to a tertiary-level academic center for suspected precocious puberty	March of 200 to June of 2021	January of 2016 to February of 2020	133 females	Incidence of CPP diagnosis in endocrinology referrals	72	61	1.3 fold-higher during COVID pandemic
Goggi G, et al. 2023 (^39^)	Retrospective cohort	Italy	Children who were referred by their Primary Care Pediatrician for suspected precocious puberty	March of 2020 to July of 2021	2014 to February of 2020	49 females	Incidence of CPP diagnosis in endocrinology referrals	30	19	3 fold-higher during COVID pandemic

**Table 2:** Positive findings in electronic devices use

Reference	Study Method	Country	Population	Exposure/Case	Comparison/Control	Sample Size, n	Outcome				
							Group	Control	Exposure	Observed changes	Electronic devices use
Stagi S, et al. 2020 (^14^)	Retrospective evaluation of medical records	Italy	Caucasian patients referred to Endocrinology Unit for CPP (group 1)	March to July of 2020	2015 to 2019 (March to July of each year)	126 females	New diagnosis of CPP	89 in total, 17/year average	37	2.17-fold increase in the number of diagnosis compared to the average of previous 5 years	Reported increase in electronic device use after lockdown
			Patients followed for untreated and slow progressive precocious puberty			22 females	Accelerated pubertal progression in previously diagnosed patients	11 in total, 2.5/year average	11	4.4-fold increase in the rate of transition from slow to fast progression precocious puberty	
Barbieri C, et al. 2022 (^18^)	Case-Control Study	Italy	Girls referred to Tertiary Center for different forms of precocious puberty	March 9th of 2020 to April 30th of 2021	January 1st of 2019 to March 8th of 2020	154 females	Incidence of CPP in a tertiary center (endocrinology referrals)	9/67 (13.43%)	17/87 (19.54%)	Risk Ratio of 1.45	Girls diagnosed with CPP during lockdown (i.e., Period 2) used more electronic devices then both period 1 controls (PC 85.5% vs. 0%, Tablet 15% vs. 0, p < 0.005) and healthy controls during lockdown (PC 85.5% vs. 73%, p < 0.005, Smartphone 29% vs. 10% p < 0.005)
Chen Y, et al. 2022 (^19^)	Cross-sectional study (Retrospective cohort)	China	Girls admitted in endocrinology ward	2020 (January to December)	2016, 2017, 2018 and 2019 (January to December)	2,802,387; no info on sex	Frequency of precocious girls admitted in endocrinology ward	106/698 (15.18%)	372/1161 (32.04%)	Risk Ratio of 2.10 (only girls)	Median value of the amount of electronic screen exposure per day was up to 3 hours
			All consultations in outpatient clinic				Incidence of precocious girls in outpatient clinic	6,547/1,611,699 (0.4%)	9270/1,188,829 (0.77%)	Risk Ratio of 1.91 (only girls)	
Chioma L, et al. 2022 (^20^)	Retrospective cohort	Italy	Patients investigated in five Italian tertiary centers of Pediatric Endocrinology	March to September of 2020	March to September of 2019		Subjects referred for suspected precocious puberty	152	338	2.22-fold increase in referrals.	Overall time spent on electronic devices was greater in the 2020 group (median 5–10 h/week, IQR (1-5 to 10-15 h) in 2019 vs. 15-20 h/week, IQR (5-10 to more than 25 h) in 2020 Significant increase of weekly device use for homework
						490 (^22^)	Incidence of CPP in tertiary centers	37/140 (26.42%)	135/328 (41.15%)	Risk Ratio 1.55 in females;	
								-	-	No difference in males;	
Fu D, et al. 2022 (^22^)	Prevalence Study & Case-Control Study	China	Girls with new-onset PP (aged 5-9 years) who visited one of 22 units between February and May 2020	February to May of 2020	February to May of 2018 and 2019	6482 females	Number of new-onset precocious puberty (CPP, PT) diagnosis in females	1100.5† (0)	4281 (0)	3.89-fold increase	Use of electronic devices for prolonged periods was Reported as one of the Main risk factors
Mutlu GY, et al. 2022 (^26^)	Case-Control Study	Turkey	Girls who had been referred to pediatric endocrinology clinics due to precocious puberty (retrospective evaluation of medical records)	March of 2020 to September of 2020	March of 2019 to March of 2020	359 females	Incidence of CPP diagnosis in endocrinology referrals due to precocious puberty	19/214 (8.8%)	42/145 (28.9%)	Risk Ratio of 3.28 during the first 6 months of pandemic	Screen time was significantly higher in the pandemic group (4.1 vs. 2.6 h/day, p < 0.001)
Chioma L, et al. 2023 (^33^)	Retrospective cohort	Italy	Patients consulting for suspected precocious or early puberty in outpatient clinic	March to September of 2020	March to September of 2019	550 females	Prevalence of suspected precocious puberty among endocrinology consultations	78/1260 (6.2%)	202/747 (27%)	Prevalence Ratio of 4.35 in 2020	Weekly time spent on electronic devices (as tablet, PC or smartphone) was greater in the 2020 group than in 2019 and 2022 groups (median >20 h/week, IQR (0) in 2020 vs. 10-15 h/week, IQR (0) in 2019 and 5-10 h/week, IQR (1-5 h/week to 10-15 h/week) in 2022; p < 0.01)
							Incidence of CPP diagnosis in endocrinology referrals	18/78 (23%)	86/202 (42.57%)	Risk Ratio of 1.85 during the lockdown	
Fava D, et al. 2023 (^38^)	Retrospective cohort	Italy	Health records of girls referred to a tertiary-level academic center for suspected precocious puberty	March of 200 to June of 2021	January of 2016 to February of 2020	133 females	Incidence of CPP diagnosis in endocrinology referrals	72	61	1.3 fold-higher during COVID pandemic	Group of girls diagnosed between March 2020 and the end of June 2021, during the COVID-19 pandemic showed a medium of 2 or more daily hours spent using electronic devices (1.946 ± 1.813 hours/day)

**Table 3:** Positive findings in BMI and auxological

Reference	Study Method	Country	Population	Exposure/Case	Comparison/Control	Sample Size, n	Outcome				
							Group	Control	Exposure	Observed changes	BMI and auxological findings
Stagi S, et al. 2020 (^14^)	Retrospective evaluation of medical records	Italy	Caucasian patients referred to Endocrinology Unit for CPP (group 1)	March to July 2020	2015 to 2019 (March to July of each year)	126 females	New diagnosis of CPP	89 in total, 17/year average	37	2.17-fold increase in the number of diagnosis compared with the average of the previous 5 years	A more advanced Tanner stage at diagnosis in exposure group
			Patients followed for untreated and slow progressive precocious puberty			22 females	Accelerated pubertal progression in previously diagnosed patients	11 in total, 2.5/year average	11	4.4-fold increase in the rate of transition from slow to fast progression of precocious puberty	
Vilella LA, et al. 2021 (^16^)	Case-control study	Spain	Patients evaluated due to premature thelarche	March to December 2020	March to December 2019	172 females	Incidence of CPP diagnosis in endocrinology referrals due to early thelarche	12/77 (16%)	25/97 (25.7%)	Risk Ratio of 1.6	Patients in exposure show increased rate of weight gain in the 6 months previous to the first consultation (31.57% vs. 12.16%)
Barbieri C, et al. 2022 (^18^)	Case-control study	Italy	Girls referred to a tertiary center for different forms of precocious puberty	March 9, 2020 to April 30, 2021	January 1, 2019 to March 8, 2020	154 females	Incidence of CPP at a tertiary center (endocrinology referrals)	9/67 (13.43%)	17/87 (19.54%)	Risk Ratio of 1.45	No difference was observed in auxological, laboratorial and radiological data, except for BMI SDS (sub-Period 2.1: -0.73 ± 1.39, sub-period 2.2 0.26 ± 0.72)
Chen Y, et al. 2022 (^19^)	Cross-sectional study (Retrospective cohort)	China	Girls admitted to an endocrinology ward	2020 (January to December)	2016, 2017, 2018 and 2019 (January to December)	2,802,387; no information on sex	Frequency of CPP girls admitted to an endocrinology ward	106/698 (15.18%)	372/1161 (32.04%)	Risk Ratio of 2.10 (girls only)	Median value of weight gain in 6 months of 2 kg during lockdown
			All consultations at an outpatient clinic				Incidence of CPP girls at an outpatient clinic	6,547/1,611,699 (0.4%)	9270/1,188,829 (0.77%)	Risk Ratio of 1.91 (girls only)	
Fu D, et al. 2022 (^22^)	Prevalence study and case-control study	China	Girls with new-onset PP (aged 5-9 years) who visited one of 22 units from February to May 2020	February to May 2020	February to May 2018 and 2019	6482 females	Number of new-onset precocious puberty (CPP, PT) diagnoses in females	1100.5† (0)	4281 (0)	3.89-fold increase	The height, BW, and BMI values in the CPP and PT groups were significantly higher than those in the control group
Itani A, et al. 2022 (^23^)	Retrospective cohort	Lebanon	Medical records of Lebanese patients referred to pediatric endocrinology clinics in multiple Lebanese regions	March 2020 to February 2021	2018 and 2019	1380 females	Incidence of CPP diagnosis in endocrinology referrals	4/964 (0.41%)	19/416 (4.6%)	Risk Ratio of 11.21.	Significantly more patients with CPP were overweight after the lockdown (10/19 patients, p < .05). Patients with precocious puberty after the lockdown weighed more than those with CPP before lockdown (increased weight mean (28.67 ± 5.46 vs. 21.2 ± 1.89, p < .05) and weight percentile (21.2 ± 1.89 vs. 74.7 ± 24.01, p < 0.05) The mean BMI was higher in the second group but not statistically significant (17.37 ± 2.39 vs. 14.85 ± 1.42, p = 0.058)
							Incidence of accelerated puberty diagnosis in endocrinology referrals	8/964 (0.83%)	6/416 (1.44%)	Risk Ratio of 1.73	
Oliveira Neto CP, et al. 2022 (^27^)	Cross-sectional study	Brazil	Girls diagnosed with precocious puberty and followed up at a pediatric endocrinology outpatient clinic	July 2020 to June 2021	March 2019 to February 2020	55 (0)	Absolute increase in diagnosis	22	33	1.5-fold increase in diagnosis	Obesity was more prevalent in the group that developed puberty during the pandemic (36.4% versus 18.2%), but without statistical significance, as was Tanner’s staging at diagnosis (p = 0.16)
Orman B, et al. 2022 (^28^)	Cross-sectional study	Turkey	57 patients who were diagnosed with CPP and started GnRH analog therapy	April 2020 to July 2020	April 2019 to July 2019	57 (54 girls, 3 boys)	Analysis of auxological, clinical, endocrine and radiologic data	27	30	Earlier CPP onset age (8.54 vs. 7.92)	Treatment was started when breast stage was T3 in 57.69% of group 1 and T2 in 75% of group 2, and a statistical difference was found between them (p = 0.006)

**Table 4:** Hormonal data positive finding

Reference	Study Method	Country	Population	Exposure/Case	Comparison/Control	Sample Size, n	Outcome				
							Group	Control	Exposure	Observed changes	Hormonal data finding
Stagi S, et al. 2020 (^14^)	Retrospective evaluation of medical records	Italy	Caucasian patients referred to Endocrinology Unit for CPP (group 1)	March to July 2020	2015 to 2019 (March to July of each year)	126 females	New diagnosis of CPP	89 in total, 17/year average	37	2.17-fold increase in the number of diagnosis compared with the average of the previous 5 years	Exposure presented elevated lab parameters (LH, E2, LH peak after LHRH test)
			Patients followed for untreated and slow progressive precocious puberty			22 females	Accelerated pubertal progression in previously diagnosed patients	11 in total, 2.5/year average	11	4.4-fold increase in the rate of transition from slow to fast progression of precocious puberty	
Chen Y, et al. 2022 (^19^)	Cross-sectional study (Retrospective cohort)	China	Girls admitted to an endocrinology ward	2020 (January to December)	2016, 2017, 2018 and 2019 (January to December)	2,802,387; no information on sex	Frequency of CPP girls admitted to an endocrinology ward	106/698 (15.18%)	372/1161 (32.04%)	Risk Ratio of 2.10 (girls only)	Serum concentrations of SHBG in the 2020 group were lower than those in 2016-2019 group (70.30 *vs.* 81.64 nmol/L) (p < .001) Peak LH/FSH ratio was higher in the 2020 group than in the 2016-2019 group (1.23 *vs.* 0.83, p < .001)
			All consultations at an outpatient clinic				Incidence of CPP girls at an outpatient clinic	6,547/1,611,699 (0.4%)	9270/1,188,829 (0.77%)	Risk Ratio of 1.91 (girls only)	
Itani A, et al. 2022 (^23^)	Retrospective cohort	Lebanon	Medical records of Lebanese patients referred to pediatric endocrinology clinics in multiple Lebanese regions	March 2020 to February 2021	2018 and 2019	1380 females	Incidence of CPP diagnosis in endocrinology referrals	4/964 (0.41%)	19/416 (4.6%)	Risk Ratio of 11.21	Reports that girls had higher hormone levels (LH, estradiol)
							Incidence of accelerated puberty diagnosis in endocrinology referrals	8/964 (0.83%)	6/416 (1.44%)	Risk Ratio of 1.73	
Umano GR, et al. 2022 (^32^)	Retrospective cohort	Italy	Female patients that attended an outpatient clinic of pediatric endocrinology due to CPP	April 2020 to April 2021	April 2017 to April 2020	69 females	Absolute increase in the number of CPP cases	11†	35	3.18-fold increase during the study period	Significant higher levels of LH, FSH, and 17-beta estradiol in CPP after/during lockdown compared to those diagnosed before
							CPP incidence rate	2%	5%	Risk Ratio of 2.5	
Chioma L, et al. 2023 (^33^)	Retrospective cohort	Italy	Patients evaluated at an outpatient clinic for suspected precocious or early puberty	March to September 2020	March to September 2019	550 females	Prevalence of suspected precocious puberty among endocrinology consultations	78/1260 (6.2%)	202/747 (27%)	Prevalence Ratio of 4.35 in 2020	Reported lower basal LH level in 2020 compared to 2022 (0.7 ± 0.98 IU/L in 2020 *vs.* 1.88 ± 1.99 IU/L in 2022, p < 0.01)
							Incidence of CPP diagnosis in endocrinology referrals	18/78 (23%)	86/202 (42.57%)	Risk Ratio of 1.85 during the lockdown	
Goggi G, et al. 2023 (^39^)	Retrospective cohort	Italy	Children who were referred by their primary care pediatricians for suspected precocious puberty	March 2020 to July 2021	2014 to February 2020	49 females	Incidence of CPP diagnosis in endocrinology referrals	30	19	3-fold increase during the COVID-19 pandemic	Delta LH value expressed as percentage was significantly lower in Post-lockdown Group than in Pre-lockdown Group (p value 0.0497)

### Rates of precocious puberty

Most studies (25 of 26) found an increase in precocious puberty during the pandemic, mainly in females. Some studies reported absolute increases, while others reported incidence rates. A total of 17 studies (^16^-^20^,^23^,^25^-^26^,^29^,^31^-^33^,^35^-^39^) reported an increase in either the prevalence or the absolute number of cases, ranging from 1.3 to 4.4 times higher when compared with pre-pandemic data. In addition, another 12 studies (^14^-^16^,^20^-^22^,^24^,^27^,^30^,^32^,^38^,^39^) found a relative increase in incidence rates of precocity (*e.g.*, CPP, accelerated puberty) ranging from 1.37 to 11.21, also compared with previous years.

Regarding sex, most studies were either conducted in the female population only or did not report information on sex, with only seven studies (^15^,^20^,^25^,^28^-^29^,^31^,^37^) reporting data on male precocity. Three studies (^21^,^25^,^37^) found higher rates of CPP in males during the exposure period (2.15-fold increase or RR 2-2.93), two studies (^15^,^20^) reported no difference, and only one study (^29^) showed a reduction in male precocity rates, but without statistical significance (RR = 0.27, p > 0.05).

### Auxological and anthropometric data

The vast majority of studies investigated the interplay between anthropometric data and the variation in rates of precocious puberty. Most studies (n = 15) found no difference in BMI, height, and weight between pandemic subjects and matched controls, except for one study (^18^) that found a positive association between BMI-SDS and the incidence of central precocious puberty (CPP) in the exposure group during a subgroup analysis.

Seven studies (^14^,^16^,^19^,^22^-^23^,^27^-^28^) found significant differences in auxological or anthropometric data between exposure groups (i.e., pandemic) and controls. Two studies (^14^,^28^) found an earlier chronological age at diagnosis of CPP in the pandemic group. One study (^16^) found increased rates of weight gain in the 6 months prior to the first consultation in pandemic children. Three studies (^22^-^23^,^27^) found that CPP girls had higher BMI and/or body weight during the pandemic compared with matched controls. On the other hand, two studies (^29^,^34^) found a negative association between BMI and CPP in their exposure groups. One study (^34^) observed a lower BMI in the exposure group in a retrospective study in Argentina, meaning that CPP children evaluated during the pandemic showed lower BMI than children assessed in the previous two years. Furthermore, one study (^29^) found lower BMI SDS in pandemic girls diagnosed with CPP compared with those diagnosed in 2019.

### Radiologic data

Eleven studies (^14^,^18^,^23^,^27^-^28^,^30^-^31^,^35^,^37^-^39^) included radiologic data in their analysis, mainly ultrasonography (USG) evaluation of uterine or ovarian length/volume or bone age assessment. The majority (n = 8) found no differences between pandemic CPP children and matched pre-pandemic controls, while the remaining studies had conflicting results. Two studies (^14^,^23^) found larger uterine and/or ovarian volumes in their exposure groups. Conversely, one study (^27^) found lower ovarian volumes in CPP girls assessed during the pandemic than in CPP girls assessed in previous years in a retrospective study of Brazilian subjects. Lastly, in terms of bone age, one study (^28^) found more advanced bone age at diagnosis in the pandemic group.

### Laboratory data

Ten studies (^14^,^18^-^19^,^22^-^23^,^27^,^32^-^33^,^35^,^37^) investigated differences in the hormonal parameters of subjects with suspected or diagnosed precocious puberty. Half of them (^18^,^27^,^33^,^35^,^37^) found no overall significant differences in laboratory data between exposure and control groups, except for a higher LH peak after LHRH test (^14^,^18^) or basal LH levels (^33^) in the exposure group. However, the other half of the studies found significant differences in laboratory parameters between pandemic and pre-pandemic CPP children. One study (^19^) found lower concentrations of SHBG and a higher LH/FSH ratio in pandemic girls, and another study (^22^) observed increased levels of kisspeptin. Lastly, three studies (^14^,^23^,^32^) found increased hormonal levels in exposure groups compared with matched controls.

### Electronic device use and lifestyle factors

Nine studies (^14^,^18^-^20^,^22^,^26^,^32^-^33^,^36^) investigated the association between the use of electronic devices and CPP rates during the pandemic. Only two studies (^32^,^36^) found no difference between screen/smartphone use by CPP girls during the pandemic and screen/smartphone use by children during previous years. In contrast, the remaining seven studies (^14^,^18^-^20^,^22^,^26^,^33^) found an overall increase in screen time during lockdown. In fact, one study (^26^) found a significant increase in daily screen time in 2020 compared with 2019 (4.1 *vs.* 2.6 h/day, p < 0.001). Furthermore, one study (^33^) observed an increase in average exposure to electronic devices, going from 5-10 hours per week to more than 25 hours per week in 2020.

Regarding other putative risk factors, seven studies (^19^-^20^,^22^,^32^-^33^,^35^-^36^) explored differences between pandemic and pre-pandemic CPP children. Three studies (^19^-^20^,^33^) observed significantly lower physical activity levels in pandemic CPP children. One study (^32^) found higher rates of sleep disorders in the exposure group, namely excessive somnolence (p = 0.049), sleep breathing disorders (p = 0.049), and sleep-wake transition disorders (p = 0.005). Moreover, this study observed that the CPP group was more likely to shift to a later bedtime (p = 0.03) during lockdown compared with controls. One study (^35^) found that children with CPP in the pandemic group had more difficulties with hyperactivity/inattention (p = 0.04). Another study (^22^) reported that the main risk factors associated with CPP during lockdown were vitamin D deficiency, obesity, consumption of processed meat, exposure to secondhand smoke, and prolonged use of electronic devices. Lastly, only one study (^36^) observed no differences in exposure to exogenous agents, physical activity, screen use, bedtime routine, or family climate between the CPP group and healthy matched controls during the pandemic.

## DISCUSSION

This scoping review evaluated 26 studies that analyzed the effect of the COVID-19 pandemic on the global incidence of CPP. All but one study reported a significant increase in the number of CPP diagnoses in girls. The number of studies that included boys was much smaller, and the results were mixed. However, the three studies that reported an increase in CPP rates in boys had the larger sample sizes. A possible explanation for the conflicting results observed in boys may be the underdiagnosis of CPP in this group. Identifying the onset of puberty in boys is challenging because testicular enlargement, which is the first sign of male puberty, is less evident compared to thelarche and menarche in girls.

A recent review on the incidence of central precocious puberty during the COVID-19 pandemic corroborates the findings in our study (^40^). The rates of precocious puberty have increased during the COVID-19 pandemic, and this is associated with factors such as the direct effect of SARS-coV-2 infection, increasing BMI of adolescents over sequential lockdowns, changes in sleep patterns, increased use of electronic devices and levels of stress, and, additionally, potential earlier detection of signs of CPP by parents and carers.

Nutritional status plays an important role in regulating pubertal onset and progression, particularly in girls (^41^). Children with higher BMI during infancy and childhood have been shown to have earlier pubertal development, and rapid BMI growth is associated with an increased risk of early puberty in girls but not in boys (^42^-^44^). Furthermore, an association of earlier menarche with overweight and obesity has been already described in girls (^45^,^46^). A recent study reported that the change in the BMI z-score of children increased approximately tenfold during the COVID-19 pandemic compared with previous years (from approximately +0.03/year to +0.34/year). This acceleration in BMI gain was observed in children of different ethnic groups, grades, and sexes, but not in those with overweight or obesity before the COVID-19 pandemic (^47^). In this review, the majority of studies investigated variations in anthropometric parameters as a proxy for nutritional status. Although some studies reported higher BMI in CPP girls during the pandemic, most studies (n = 15) found no significant association between weight and/or BMI and rates of precocious puberty. These controversial results can be partially explained by biases inherent in retrospective study designs, which often affect the accuracy of their findings. One significant issue is selection bias, as these studies frequently rely on existing records and data that may not accurately represent the general population. When certain demographic or clinical groups are overrepresented, it can skew the results. Another concern is recall bias, which arises in studies where data is collected through questionnaires or interviews. Participants may struggle to accurately remember or report their past BMI or nutritional status, leading to potential misclassification of the data. Additionally, confounding variables can complicate the understanding of the relationship between BMI and precocious puberty. Multiple factors, such as socioeconomic status, genetic predispositions, and environmental exposures, can influence both BMI and the onset of puberty. If these confounders are not adequately controlled for, researchers may draw erroneous conclusions about the association between the two. Measurement bias is also a critical factor to consider, as variability in how BMI is measured can introduce inconsistencies in the data. For instance, some studies may rely on self-reported measurements while others utilize clinical assessments, making it challenging to compare results across studies. Finally, publication bias can impact the perceived relationship between BMI and precocious puberty. Studies that identify significant associations are more likely to be published than those reporting null results, which can lead to an overestimation of the true relationship between the two variables. Together, these biases highlight the need for careful consideration and methodological rigor in studies examining the connections between BMI and precocious puberty.

Another factor contributing to the increase in cases of CPP was the significant reduction in time spent in physical activity during the pandemic. This lack of physical activity may have contributed to worsening body composition, decreased muscle mass, and increased fat deposition (^20^). In addition, one study showed that intense physical activity is associated with a delay in the age of onset of menarche (^48^).

Another important aspect is the impact of social stress on puberty, as stressful events are believed to accelerate sexual maturation (stress acceleration hypothesis) (^3^). Possible mediators of the association between stress/anxiety and pubertal onset include increased expression of the gamma-aminobutyric acid (GABA) receptor on the dendrites of CA1 pyramidal cells and increased catecholaminergic activity in the hippocampus (^49^,^50^). Previous studies have shown that exposure to early life adversity and traumatic stressful events is associated with earlier puberty and age at menarche, and the experience of COVID-19 quarantine was a particularly stressful experience for parents and children (^51^-^53^). A recent elegant study investigated children’s mental health during the COVID-19 pandemic across quarantine and periods of less severe social isolation in Germany. Parental stress was a risk factor that amplified the negative effects of the pandemic on children’s psychological well-being, and while children’s emotional well-being recovered during periods of less severe social isolation, their family-related well-being steadily decreased over the course of the pandemic (^54^). Another study showed that anxiety in prepubescent girls was associated with the onset of early puberty, regardless of maternal anxiety, BMI, ethnicity, and maternal education (^55^).

Other factors have also been hypothesized to contribute to the increase in CPP cases during the pandemic, such as the direct effect of SARS-CoV-2, changes in sleep and reduction in melatonin secretion, increased digital screen time, exposure to endocrine disrupting chemicals, changes in the microbiota, and vitamin D deficiency (^10^,^11^). As expected, most studies investigating changes in screen time found an overall increase in the general use of electronic devices during the pandemic. The main use was for school activities, but also for entertainment or in the hours before sleep (^14^). In fact, one study (^32^) reported statistically significantly higher rates of sleep disturbance in CPP girls. Moreover, a study conducted in China with a large sample size (n = 6,482) (^22^) reported the presence of vitamin D deficiency, consumption of processed meat, exposure to secondhand smoke, prolonged use of electronic devices, and obesity as the main risk factors for central precocious puberty and premature thelarche. As for the direct effect of SARS-CoV-2, it is known that GnRH neurons in the hypothalamus share a common embryonic origin with olfactory bulb neurons; therefore, a direct action of the virus on both these neurons could potentially trigger the onset of puberty, but this association remains to be confirmed in future dedicated studies (^11^). Finally, compared with the pre-pandemic period, positive associations were found among children with CPP in terms of the use of electronic devices, some of which were related to sleep disorders.

Some limitations of our review must be considered. First, the majority of the studies only provided data on the number of diagnosed cases, without providing information on the exposed population or the number of evaluations during the period. Therefore, the increase in the number of diagnoses may have occurred because of a greater demand for care during the pandemic due to increased parental observation. Second, the associations found as possible risk factors for precocious puberty cannot establish a causal relationship, but only raise hypotheses for further investigation. Third, there was a predominance of studies in Italy compared with other countries. However, the results in the population of girls were consistent worldwide. Despite these limitations, we believe that we have been able to effectively find and summarize the current data on the effect of the COVID-19 lockdown on the incidence of CPP.

In conclusion, the systematic review of the studies published so far has demonstrated a consistent increase in the incidence of precocious puberty in girls during the COVID-19 pandemic, but not in boys. Possible causes for this increased incidence include increased screen time, reduced physical activity, psychological stress, changes in diet and sleep habits, and the direct effects of SARS-CoV-2. Continued monitoring of the incidence of CPP will make it possible to determine whether this phenomenon will continue after the pandemic.
